# Paediatric medical emergency calls to a Danish Emergency Medical Dispatch Centre: a retrospective, observational study

**DOI:** 10.1186/s13049-017-0470-1

**Published:** 2018-01-05

**Authors:** Kasper Andersen, Søren Mikkelsen, Gitte Jørgensen, Stine Thorhauge Zwisler

**Affiliations:** 1 Department of Clinical Research, University of Southern Denmark, Odense, Denmark; 2 Department of Anaesthesiology & Intensive Care, Odense University Hospital, Odense, Denmark; 3 Department of Anaesthesiology & Intensive Care, Mobile Emergency Care Unit, Odense University Hospital, Odense, Denmark; 4 Department of Clinical Medicine, Centre for Pre-hospital and Emergency Research, Aalborg University, Aalborg, Denmark; 5 Emergency Medical Dispatch Centre, Region of Southern Denmark, Odense, Denmark

**Keywords:** Emergency medical dispatching, Criteria-based dispatch, Paediatric medical emergency

## Abstract

**Background:**

Little is known regarding paediatric medical emergency calls to Danish Emergency Medical Dispatch Centres (EMDC). This study aimed to investigate these calls, specifically the medical issues leading to them and the pre-hospital units dispatched to the paediatric emergencies.

**Methods:**

We performed a retrospective, observational study on paediatric medical emergency calls managed by the EMDC in the Region of Southern Denmark in February 2016. We reviewed audio recordings of emergency calls and ambulance records to identify calls concerning patients ≤ 15 years. We examined EMDC dispatch records to establish how the medical issues leading to these calls were classified and which pre-hospital units were dispatched to the paediatric emergencies. We analysed the data using descriptive statistics.

**Results:**

Of a total of 7052 emergency calls in February 2016, 485 (6.9%) concerned patients ≤ 15 years. We excluded 19 and analysed the remaining 466. The reported medical issues were commonly classified as: “seizures” (22.1%), “sick child” (18.9%) and “unclear problem” (12.9%). The overall most common pre-hospital response was immediate dispatch of an ambulance with sirens and lights with a supporting physician-manned mobile emergency care unit (56.4%). The classification of medical issues and the dispatched pre-hospital units varied with patient age.

**Discussion:**

We believe our results might help focus the paediatric training received by emergency medical dispatch staff on commonly encountered medical issues, such as the symptoms and conditions pertaining to the symptom categories “seizures” and “sick child”. Furthermore, the results could prove useful in hypothesis generation for future studies examining paediatric medical emergency calls.

**Conclusion:**

Almost 7% of all calls concerned patients ≤ 15 years. Medical issues pertaining to the symptom categories “seizures”, “sick child” and “unclear problem” were common and the calls commonly resulted in urgent pre-hospital responses.

**Electronic supplementary material:**

The online version of this article (10.1186/s13049-017-0470-1) contains supplementary material, which is available to authorized users.

## Background

The staff at Emergency Medical Dispatch Centres (EMDCs) play a key role in the management of out-of-hospital medical emergencies [1]. They assess citizens’ medical emergency calls, dispatch pre-hospital units to treat and transport patients and provide medical advice and first aid instructions to callers. The staff’s assessments are based on telephone triage performed during the medical emergency call and such triage may be a challenging task [2]. Precise and exact triage is vital, as undertriage may result in inadequate first aid instructions and the dispatch of insufficient pre-hospital resources, which may lead to inferior outcomes for certain patients [3–5]. On the other hand, overtriage may result in inexpedient allocation of pre-hospital resources, reduced ambulance availability and emergency department overcrowding [6].

Various aspects of emergency medical dispatch have previously been examined in Scandinavian studies, including causes for medical emergency calls [7, 8], difficulties and barriers associated with telephone triage [1, 2], and the use of criteria-based dispatch protocols [9, 10]. Nevertheless, little attention has been paid to paediatric emergencies. This is unfortunate, as robust data on paediatric medical emergency calls could help focus EMDC training procedures on commonly reported medical issues, evaluate the adequacy of pre-hospital responses to paediatric emergencies and suggest priorities for future research in the field of pre-hospital paediatric care. An enquiry into paediatric medical emergencies attended by physicians seems particularly prudent, as dispatch criteria for physician-provided pre-hospital critical care have been named an important research topic in a European consensus report [11].

Consequently, the aim of this study was to investigate paediatric medical emergency calls to a Danish EMDC. Primarily, we wanted to examine how the EMDC staff classified the medical issues leading to these calls and which pre-hospital units were dispatched to the paediatric emergencies. Secondarily, we wanted to examine if classification and dispatched units varied with patient age.

## Methods

### Study design

We performed a retrospective, observational study on paediatric medical emergency calls managed by the EMDC in the Region of Southern Denmark in February 2016. This EMDC covers 1,210,000 people, roughly 20% of the Danish population, living in both rural and urban areas [12].

### Setting

In Denmark, citizens use the national emergency number, 1–1-2, to request assistance from the various emergency services. Callers reporting medical emergencies are diverted to the regional EMDC, where experienced and specially trained nurses, emergency medical technicians (EMTs) and paramedics assess and prioritise the incoming calls using a criteria-based dispatch protocol, the so-called “Danish Index for Emergency Care” [10]. The dispatch protocol is composed of 37 symptom categories, each divided into five priority levels named level A through level E. Level A describes life-threatening or potentially life-threatening issues, which call for the immediate dispatch of an ambulance with sirens and light. Level B describes urgent issues, which call for the immediate dispatch of an ambulance but without sirens and light. Level C describes non-urgent issues, which call for the dispatch of an ambulance within a given time frame. Level D describes non-urgent issues, which call for supine patient transport but not necessarily treatment or observation during transportation. Level E describes the least urgent issues, which do not call for an ambulance, but rather medical advice or referral of the patient to a general practitioner or a similar health-care authority [2, 10]. Each priority level encompasses multiple dispatch codes pertaining to more specific symptoms and conditions [10]. An overview of the 37 symptom categories are presented in Additional file 1: Figure S1.

For each call, the EMDC staff classify the primary medical issue according to one of the dispatch protocol’s symptom categories and assign a fitting dispatch code which describes the nature and urgency of the emergency. The protocol subsequently translates the assigned dispatch code to a pre-hospital response suitable for the emergency and medical advice or first aid instructions for the caller [10]. The basic pre-hospital unit is an ambulance manned by two EMTs, but units with more advanced medical qualifications can also be dispatched to assist the ambulance crews. These include: paramedics, anaesthesiologist-manned Mobile Emergency Care Units (MECU) and Helicopter-based Emergency Medical Services (HEMS) [13, 14].

The EMDC in the Region of Southern Denmark stores all emergency calls as audio recordings. Furthermore, the EMDC staff manually fills out dispatch records, which state patient information obtained during the call, the assigned dispatch code and the dispatched pre-hospital units. The dispatched pre-hospital units also file records.

### Participants

Medical emergency calls concerning patients ≤ 15 years were eligible for inclusion. We identified these calls by manually reviewing the audio recordings of all emergency calls managed by the EMDC during the study period. It was possible to identify relevant calls this way, as the EMDC staff usually question the caller regarding patient’s age. If we suspected a call to be eligible for inclusion from the context of the audio recording, but the patient’s age was not explicitly stated, we examined the corresponding ambulance record. If we could not establish the patient’s age after thoroughly reviewing these two data sources, we did not include the call for analysis. This procedure was chosen to ensure that even if the dispatch record is not completely precise and if the contact did not elicit an ambulance – and thus no ambulance record – the call was included into the study.

We applied two exclusion criteria to calls concerning patients ≤ 15 years. Firstly, we excluded calls where a previous caller had already reported the same emergency to the EMDC. Secondly, we excluded calls where a caller contacted the EMDC again while waiting for the dispatched pre-hospital unit(s) to arrive. We divided the included calls into three groups based on patient age: < 1 year, 1–5 years and 6–15 years.

### Variables and data analysis

We reviewed the audio recordings and ambulance records to establish the age and gender of the patients. We reviewed the dispatch records to establish the assigned dispatch codes and pre-hospital units. When we found variables to be missing, we labelled them as such.

We analysed the data using descriptive statistics. The analyses were performed with SPSS (IBM Corp. Released 2016. IBM SPSS Statistics for Windows, Version 24.0. Armonk, NY: IBM Corp.). For proportions, we calculated 95% confidence intervals (95% CI) based on a binomial distribution.

## Results

### Contact and patient characteristics

**Fig. 1:**
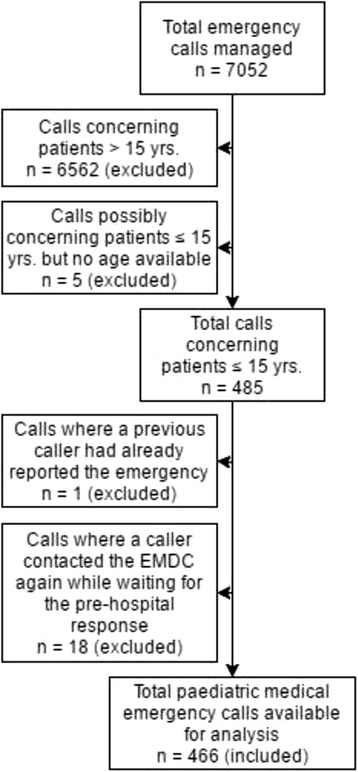
Data collection flowchart. Flowchart showing the data collection process

The EMDC managed 7052 medical emergency calls during February 2016. We identified 459 calls concerning patients ≤ 15 years by reviewing audio recordings and 26 by reviewing ambulance records. We suspected five further calls to concern eligible patients, but we did not include these as we could not establish the age of these patients. Accordingly, 485 (6.9%) of all calls managed by the EMDC during the study period concerned paediatric patients. We excluded 19 of these in adherence to our exclusion criteria and analysed the remaining 466. Figure 1 presents an overview of the data collection process.

Calls concerning males accounted for 51.9% (*n* = 242) and calls concerning females for 47.9% (*n* = 223). For one call, we could not verify the patient’s gender. The median age was 4 years (range 0–15 years). Fifty-six (12.0%) calls concerned patients < 1 year, 208 (44.6%) concerned patients aged 1–5 years and 202 (43.4%) concerned patients aged 6–15 years.

### Classification of medical issues

The EMDC staff frequently classified the issues leading to the paediatric medical emergency calls according to the following symptom categories: “seizures” (22.1%), “sick child” (18.9%), “unclear problem” (12.9%), “ordered mission” (i.e. ambulance runs ordered by general practitioners) (12.7%) and “accident” (9.2%). Accordingly, over 75% of the reported issues pertained to these five symptom categories. We were unable to locate a valid dispatch code for 29 (6.2%) of the calls. Of all contacts, 71 (15.2%) regarded various trauma patients.

Table 1 presents how the medical issues leading to the emergency calls were classified, including results stratified for patient age. The table seems to indicate that the commonly reported medical issues varied with patient age. For patients < 1 year, issues pertaining to “sick child” were most common (37.5%), whereas it was issues pertaining to “seizures” for patients aged 1–5 years (37.0%) and issues pertaining to “unclear problem” for patients aged 6–15 years (17.8%).

**Table 1:** Classification of reported medical issues

Symtom category	All		< 1 yr.		1–5 yrs.		6–15 yrs.	
	n	% (95%CI)	n	% (95%CI)	n	% (95%CI)	n	% (95%CI)
*Seizures*	103	22.1 (18.4–26.2)	7	12.5 (5.2–24.1)	77	37.0 (30.4–44.0)	19	9.4 (5.8–14.3)
*Sick child*	88	18.9 (15.4–22.7)	21	37.5 (24.9–51.5)	49	23.6 (18.0–29.9)	18	8.9 (5.4–13.7)
*Unclear problem*	60	12.9 (10.0–16.3)	9	16.1 (7.6–28.3)	15	7.2 (4.1–11.6)	36	17.8 (12.8–23.8)
*Ordered mission*	59	12.7 (9.8–16.0)	9	16.1 (7.6–28.3)	27	13.0 (8.7–18.3)	23	11.4 (7.4–16.6)
*Accidents*	43	9.2 (6.8–12.2)	4	7.1 (2.0–17.3)	10	4.8 (2.3–8.7)	29	14.4 (9.8–20.0)
*Minor wound, fracture, injury*	13	2.8 (1.5–4.7)	.	.	3	1.4 (0.3–4.2)	10	5.0 (2.4–8.9)
*Traffic accident*	12	2.6 (1.3–4.5)	1	1.8 (0.0–9.6)	3	1.4 (0.3–4.2)	8	4.0 (1.7–7.7)
*Breathing problems*	12	2.6 (1.3–4.5)	2	3.6 (0.4–12.3)	4	1.9 (0.5–4.9)	6	3.0 (1.1–6.4)
*Alcohol, poisoning, drugs*	9	1.9 (0.9–3.6)	.	.	.	.	9	4.5 (2.1–8.3)
*Foreign body in airway*	5	1.1 (0.4–2.5)	1	1.8 (0.0–9.6)	3	1.4 (0.3–4.2)	1	0.5 (0.0–2.7)
*Ear, nose, throat*	5	1.1 (0.4–2.5)	1	1.8 (0.0–9.6)	2	1.0 (0.1–3.4)	2	1.0 (0.1–3.5)
*Psychiatry, suicide*	4	0.9 (0.2–2.2)	.	.	.	.	4	2.0 (0.5–5.0)
*Allergic reaction*	3	0.6 (0.1–1.9)	.	.	.	.	3	1.5 (0.3–4.3)
*Non-traumatic bleeding*	3	0.6 (0.1–1.9)	.	.	1	0.5 (0.0–2.7)	2	1.0 (0.1–3.5)
*Burn or electrical injury*	3	0.6 (0.1–1.9)	.	.	1	0.5 (0.0–2.7)	2	1.0 (0.1–3.5)
*Chest pain, heart disease*	3	0.6 (0.1–1.9)	.	.	.	.	3	1.5 (0.3–4.3)
*Stomach or back pain*	3	0.6 (0.1–1.9)	.	.	1	0.5 (0.0–2.7)	2	1.0 (0.1–3.5)
*Diabetes*	2	0.4 (0.1–1.5)	1	1.8 (0.0–9.6)	.	.	1	0.5 (0.0–2.7)
*Poisoning in child*	2	0.4 (0.1–1.5)	.	.	1	0.5 (0.0–2.7)	1	0.5 (0.0–2.7)
*Impaired consciousness*	2	0.4 (0.1–1.5)	.	.	.	.	2	1.0 (0.1–3.5)
*Unconscious adult*	1	0.2 (0.0–1.2)	.	.	1	0.5 (0.0–2.7)	.	.
*Unconscious child*	1	0.2 (0.0–1.2)	.	.	1	0.5 (0.0–2.7)	.	.
*Headache*	1	0.2 (0.0–1.2)	.	.	.	.	1	0.5 (0.0–2.7)
*Missing dispatch code*	29	6.2 (4.2–8.8)	.	.	9	4.3 (2.0–8.1)	20	9.9 (6.2–14.9)
Total	466	100	56	100	208	100	202	100

All calls (*n* = 466) distributed according to medical issue classification and age group. Percentages rounded to one decimal

Additional file 2: Table S1 presents all assigned dispatch codes, including frequencies and proportions. As such, it provides a more detailed overview of the reported medical issues.

### Dispatched pre-hospital units

An ambulance was dispatched to 87.2% of all paediatric emergencies. Ambulances with lights and sirens with additional MECU support accounted for more than half of the responses to the calls. For details, see Table 2, which presents the pre-hospital units dispatched in response to the paediatric medical emergency calls.

**Table 2:** Dispatched pre-hospital units

Dispatched pre-hospital units	All		< 1 yr.		1–5 yrs.		6–15 yrs.	
	n	% (95%CI)	n	% (95%CI)	n	% (95%CI)	n	% (95%CI)
*Ambulance with MECU, level A*	263	56.4 (51.8–61.0)	44	78.6 (65.6–88.4)	152	73.1 (66.5–79.0)	67	33.2 (26.7–40.1)
*Ambulance, level A*	64	13.7 (10.7–17.2)	3	5.4 (1.1–14.9)	14	6.7 (3.7–11.0)	47	23.3 (17.6–29.7)
*Ambulance, level B*	64	13.7 (10.7–17.2)	4	7.1 (2.0–17.3)	13	6.3 (3.4–10.5)	47	23.3 (17.6–29.7)
*No ambulance, level E*	59	12.7 (9.8–16.0)	5	8.9 (3.0–19.6)	23	11.1 (7.1–16.1)	31	15.3 (10.7–21.1)
*Ambulance with PM, level A*	15	3.2 (1.8–5.3)	.	.	5	2.4 (0.8–5.5)	10	5.0 (2.4–8.9)
*Ambulance, level C*	1	0.2 (0.0–1.2)	.	.	1	0.5 (0.0–2.7)	.	.
Total	466	100	56	100	208	100	202	100

All calls (*n* = 466) distributed according to dispatched pre-hospital units and age group. Emergency priority level is also shown. Percentages rounded to one decimal. *MECU* Mobile Emergency Care Unit, *PM* Paramedic

## Discussion

### Main findings

This retrospective, observational study aimed to examine paediatric medical emergency calls to a Danish EMDC. Our results suggest that the EMDC staff commonly classified the medical issues leading to these calls as “seizures”, “sick child” and “unclear problem”, and that the calls generally triggered urgent pre-hospital responses. Issue classification and dispatched pre-hospital units both seem to vary with patient age.

### Results in relation to existing literature

Almost 7% of the calls managed by the EMDC during the study period concerned paediatric patients. This is comparable to findings in other studies [15–17].

A substantial proportion of the medical issues leading to these calls were classified as “seizures” and this was particularly the case for calls concerning patients aged 1–5 years. As has previously been suggested, this finding might reflect the age-peak of febrile seizures [17, 18]. The results presented in Additional file 2 seem to support this notion, since many of assigned dispatch codes from the “seizures” symptom category appear to have concerned this condition. Our results suggest a somewhat higher prevalence of calls concerning seizures compared to similar international studies [15–18]. This might be attributed to our study period, as febrile seizures appear to be more common in the winter months [19]. However, it seems unlikely that this alone could cause the observed differences, as our study population had a considerable size.

The symptom category “sick child” pertains to a broad spectrum of symptoms and conditions. The dispatch codes presented in Additional file 2 suggest that many of the issues classified according to this category concerned breathing problems. This is consistent with previous findings, as respiratory problems seem to be a common cause for emergency medical service activation [15–17, 20]. However, the prevalence of calls concerning breathing problems in our study seems slightly lower than previously reported. This discrepancy might be attributed to the structure of the Danish dispatch protocol, as dispatch codes pertaining to respiratory problems and abnormal breathing are divided between multiple symptom categories, including “sick child”, “breathing problems” and “allergic reaction”.

Other studies have reported that traumatic injuries constitute 27–46% of all paediatric medical emergencies [15–17]. As such, we were surprised to find that only 15.2% of the medical issues reported in this study were classified according to one of the dispatch protocol’s trauma categories; “accidents”, “minor wound, fracture, injury”, “burn and electrical injuries” and “traffic accidents”. As with the high prevalence of seizures, the reason for this somewhat unexpected discrepancy might be our study period, as traumatic injuries in children appear to be more common in the summer [21]. However, it seems unlikely that we would see a doubling of the trauma incidence in other months in our study population. Another explanation, which is not related to the study period, may be found in contacts concerning minor trauma. These contacts, where the caller may be advised by the EMDC staff to contact the emergency department directly, are not categorised as trauma calls, but are instead assigned the dispatch code E05.02, “referred to other solution”. Lastly, the safety of Danish bicyclist is a topic that has received much attention in recent years – including campaigns directed at making cycling safer for children [22]. It is possible that the focus on traffic safety has led to a lower occurrence of traffic related trauma among Danish children.

Our results suggest that 73.3% of the paediatric medical emergency calls resulted in a priority level A pre-hospital response, i.e. the response reserved for life-threatening or potentially life-threatening issues, and that a MECU was dispatched to 56.4% of the emergencies. As Table 2 suggests, the emergencies concerning younger children generally resulted in more urgent and advanced pre-hospital responses than emergencies concerning older children. Our results are strikingly different from the 3.5% and 4% reported in a similar study recently conducted in Helsinki [15]. However, other studies comparing medical dispatch procedures in Denmark and Finland have reported similar discrepancies and have primarily pointed to differences between the two countries’ dispatch protocols and emergency medical services as plausible explanations for these contradictory findings [9, 13, 14].

Previous studies have found that 28.7–51.4% of all Danish medical emergency calls result in a priority level A response, depending on the study design [8, 10, 14]. As such, it seems that the paediatric patients in our study generally received more urgent pre-hospital responses compared to the overall Danish population. There are several possible explanations for this. Firstly, the EMDC staff in the Region of Southern Denmark adhere to a specific guideline stating that emergencies concerning patients under 2 years should receive a priority level A response with MECU support. However, this alone does not seem to account for the substantial differences observed. Another explanation might be that the EMDC staff find it more difficult to assess paediatric emergencies and consequently tend to err on the side of caution by deliberately over-triaging these patients. Previous studies have reported that emergency medical service providers find assessments and decision-making harder in paediatric patients, so it seems plausible that EMDC staff should experience similar problems and act accordingly [23]. In contrast, it has previously been shown that EMDC staff may be able to identify low acuity cases without the risk of committing undertriage [24]. Our finding, which raises suspicion of over triage thus highlights the need for further education of the EMDC staff and a closer assurance of the quality in triaging these patients. In order to reduce the apparent overtriage of the prehospital services, a system providing more extended on-site assistance – for example by prehospital trained physicians could be implemented.

### Strengths and limitations

One strength was the broad demographic spectrum covered by our catchment area. The primary strength of this study, however, was the inclusion strategy, which we believe was efficient in including paediatric medical emergency calls. As our results show, it was appropriate to manually review two separate data sources, audio recordings and ambulance records to identify eligible contacts, as the data sources complemented each other. We decided on this inclusion strategy as the EMDC staff sometimes do not have the opportunity to state the age of the patient in the dispatch record – for instance, the patient’s age might be unknown to the caller. Consequently, searching *only* the dispatch records could have resulted in the inappropriate exclusion of relevant calls and possibly induced selection bias. Furthermore, the EMDC software used in the Region of Southern Denmark is not very efficient at searching for patients based on age, so it is likely that we would have missed patients, had we used this approach.

The main limitation of the study was the single centre design. As we extracted data from a single EMDC, our results might reflect the triage and dispatch culture at this centre only, which would limit the external validity of the results. Another limitation was the short study period, as we chose to include contacts from only 1 month. Our prioritisation of completeness of data over a longer study period might have made our results vulnerable to seasonal variation in disease patterns. Finally, we were limited by the 29 missing dispatch codes. The need for the EMDC staff to manually fill out the dispatch records might explain the missing data, as they may rush or interrupt this process in the case of another emergency call. Should this be the explanation, we believe that a classification bias would be unlikely as there would be no pattern to the data missing.

### Implications of results and future studies

We believe that our results have certain implications for the paediatric training offered to the Danish EMDC staff. This training should evidently prepare the staff to accurately identify serious medical conditions, e.g. cardiac arrest and to provide verbal first aid instructions to callers reporting such emergencies. However, it seems prudent that the training should also focus on commonly encountered medical issues. Taking our results into consideration, EMDC staff should be confident in identifying symptoms and conditions pertaining to the symptom categories “seizures” and “sick child”, particularly febrile seizures and breathing problems, and in providing pre-arrival first aid instructions to assist callers reporting these emergencies. We consider these implications important for the planning of EMDC training procedures, as an emphasis on the most important and the most common medical issues could be instrumental in preparing the staff for managing paediatric medical emergency calls.

Our study was not designed to assess the accuracy of the assigned dispatch codes or the adequacy of the pre-hospital responses. However, we believe our results might be useful for hypothesis generation in future studies examining these topics. The keeping of ambulance records opens the possibility that the prehospital personnel may grade, or evaluate, the dispatch codes, but this is not done systematically at present. Methodologically, future studies should try to include more than a single EMDC to increase the catchment area and improve external validity. Furthermore, future studies should expand the study period to reduce possible confounders originating from seasonal variation in disease patterns.

## Conclusion

This retrospective, observational study on medical emergency calls to a Danish EMDC found that paediatric emergencies accounted for almost 7% of all calls. The EMDC staff commonly classified the medical issues leading to these calls as “seizures”, “sick child” and “unclear problem” and dispatched urgent pre-hospital units to the paediatric emergencies, often with MECU support. Medical issue classification and dispatched pre-hospital units both seem to have varied with patient age. We believe these results could be useful in the planning of the paediatric training received by Danish EMDC staff and in hypothesis generation for future studies examining paediatric medical emergency calls.

## Additional files

Additional file 1: Figure S1. Overview of the categories in the Danish Index for Emergency Care. A figure listing the 37 different categories in the criteria-based dispatch protocol used by the Danish EMDCs. (PDF 449 kb)

Additional file 2: Table S1. Assigned dispatch codes. A table showing the assigned dispatch code for every included paediatric medical emergency call. (XLSX 20 kb)

## Abbreviations

EMDC: Emergency medical dispatch centre
EMT: Emergency medical technicians
HEMS: Helicopter-based emergency medical service
MECU: Mobile emergency care unit
