# Supplementary material for: Paediatric medical emergency calls to a Danish Emergency Medical Dispatch Centre: a retrospective, observational study
Source: Scand J Trauma Resusc Emerg Med. 2018 Jan 5;26:2. doi: 10.1186/s13049-017-0470-1 (PMC5756442; doi:10.1186/s13049-017-0470-1)
Supplement: Supplementary file 1 — Overview of the categories in the Danish Index for Emergency Care. A figure listing the 37 different categories in the criteria-based dispatch protocol used by the Danish EMDCs. (PDF 449 kb) [file 13049_2017_470_MOESM1_ESM.pdf]

**Additional file 1: Figure S1: Overview of the categories in the Danish Index for Emergency Care**

The main symptom categories in the Danish Index for Emergency Medical Care are presented in the table below.

| | **Category** | **Note** |
|---|---|---|
| *1* | Unconscious adult | |
| *2* | Unconscious child | |
| *3* | Foreign body in airway | |
| *4* | Large scale accident | |
| *5* | Ordered mission | Includes referrals and ambulance runs ordered by physicians. |
| *6* | Unclear problem | |
| *7* | Allergic reaction | |
| *8* | Non-traumatic bleeding | |
| *9* | Burn or electricity injury | |
| *10* | Chest pain, heart disease | |
| *11* | Diabetes | |
| *12* | Drowning | |
| *13* | Diving accident | |
| *14* | Animal bite or insect bite | |
| *15* | Fever | |
| *16* | Poisoning in child | |
| *17* | Childbirth | |
| *18* | Gynaecology, pregnancy | |
| *19* | Headache | |
| *20* | Skin disease, rash | |
| *21* | Hypo- and hyperthermia | |
| *22* | Exposure to chemicals and gases | |
| *23* | Seizures | |
| *24* | Stomach or back pain | |
| *25* | Possible death | |
| *26* | Impaired consciousness | |
| *27* | Psychiatry, suicide | |
| *28* | Breathing problems | |
| *29* | Alcohol, poisoning, drugs | |
| *30* | Sick child | Broad category pertaining particularly to young children. |
| *31* | Minor wound, fracture, injury | |
| *32* | Traffic accident | |
| *33* | Accidents | |
| *34* | Urinary system | |
| *35* | Violence, abuse | |
| *36* | Ear, nose, throat | |
| *37* | Eye | |
